# Effect of whey protein‐ and xanthan‐based coating on the viability of microencapsulated *Lactobacillus acidophilus* and physiochemical, textural, and sensorial properties of yogurt

**DOI:** 10.1002/fsn3.2398

**Published:** 2021-06-10

**Authors:** Mina Khorshidi, Ali Heshmati, Mehdi Taheri, Mostafa Karami, Reza Mahjub

**Affiliations:** ^1^ Department of Nutrition and Food Safety School of Medicine Nutrition Health Research Center Hamadan University of Medical Sciences Hamadan Iran; ^2^ Faculty of Food Science and Technology Bu‐Ali Sina University of Hamedan Hamedan Iran; ^3^ Department of Pharmacology and Toxicology School of Pharmacy, Medicinal Plants and Natural Products Research Center Hamadan University of Medical Sciences Hamadan Iran

**Keywords:** dairy products, *Lactobacillus acidophilus*, probiotic, viability, yogurt

## Abstract

The goal of this study was to investigate the viability of microencapsulated *and coated Lactobacillus acidophilus* in yogurt during storage in a refrigerator for 28 days and in simulated gastrointestinal conditions. Furthermore, the effect of the microencapsulated and coated *L*. *acidophilus* on the physicochemical, textural, and sensory properties of yogurt was assessed. *Lactobacillus* *acidophilus* was microencapsulated in sodium alginate and coated with xanthan and/or whey protein. The coating led to the increase in the microcapsule diameter and the microencapsulation yield, while it led to the decreased moisture and water activity (aw) of the microcapsule. The survival of *L. acidophilus* microcapsule coated with whey protein and xanthan in yogurt during storage and exposure to simulated gastrointestinal conditions was significantly increased. Compared with free bacteria, the *L. acidophilus* microcapsule coated with whey protein and xanthan had the increased viability in yogurt until 2.16 log CFU/g during storage and 3.52 log CFU/g in simulated gastrointestinal conditions. After the 28th day of storage, a significant difference between the acidity and pH of yogurt containing coated and microencapsulated *L. acidophilus* and control yogurt was not observed. However, yogurt containing free *L. acidophilus* had lower pH and higher acidity and showed a significant difference (*p* < .05) with other samples. Although the coating of *L. acidophilus* microcapsule did not affect the sensory properties and gumminess of yogurt, it increased the firmness, adhesiveness, and viscosity of this product and caused a significant decrease in syneresis and cohesiveness. In general, the application of whey protein and xanthan coating on *L. acidophilus* microcapsule surface could increase the viability of this probiotic in yogurt during storage and in simulated gastrointestinal conditions and improve the texture attributes of yogurt.

## Graphical abstract

The influence coating with xanthan and/or whey protein and microencapsulation with sodium alginate on Lactobacillus acidophilus viability in yogurt during storage and under simulated gastrointestinal conditions was investigated. In addition, the effect of microencapsulated and/or coated probiotic on the physicochemical, textural, and sensory properties of yogurt was assessed. Microencapsulation and coating of L. acidophilus led to the significant increase in the viability of the probiotic in yogurt during storage and under simulated gastrointestinal conditions and improved the texture attributes of the product.

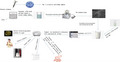

## 1 INTRODUCTION

Nowadays, there is an increased demand for functional foods such as probiotics (Afzaal et al., 2019). Probiotics are defined as “live microorganisms which when administered in adequate amounts confer a health benefit on the host” (Hill et al., 2014). They are marketed in several ways, such as dairy (Fysun et al., 2019) and non‐dairy products (Kandylis et al., 2016). The International Dairy Federation (IDF) recommends a minimum viable probiotic concentration of 10^6^–10^7^ colony‐forming unit (CFU)/g at the end of the shelf life of the product to exert therapeutic effects (IDF, 1992). To impart beneficial health effects on the host, probiotics must maintain their viability during food production, processing, and storage and pass through the gastrointestinal tract (Khorshidian et al., 2020; de Oliveira et al., 2014; Tripathi & Giri, 2014).

Microencapsulation of probiotics is suitable method for the improved survival of these bacteria in foodstuffs and the gastrointestinal tract as well as their release at a controlled rate (Afzaal et al., 2020). Microencapsulation is defined as the process of entrapment/enclosure of microorganisms within proper hydrocolloid to separate them from the surrounding environment (Afzaal et al., 2020; Krasaekoopt et al., 2004; Ramos et al., 2018). The materials used for the microencapsulation of probiotics should be of food grade and safe (Nedovic et al., 2011; Wandrey et al., 2010). Sodium alginate is widely used for the microencapsulation of probiotics due to its cost‐effectiveness, easy use, non‐toxicity, biocompatibility, and digestibility. However, sodium alginate is not stable in the presence of anti‐gel cations or chelating agents. In addition, the alginate microcapsules are sensitive to low pH and rapidly releases microencapsulated components (Iravani et al., 2015). On the other hand, alginate‐based microcapsules have porous networks and could not protect probiotics well against adverse environment conditions (Ramos et al., 2018). The application of coating or secondary layer on alginate‐based microcapsule surface could resolve the mentioned defects. The different materials utilized for coting include polycations, such as chitosan or poly‐amino acids, proteins, and gums (Gbassi et al., 2009, Yao et al., 2020). In some cases, the mentioned materials themselves were applied for microcapsule production. The coating creates an additional membrane on alginate‐based microcapsule surface and improves its performance (Ramos et al., 2018).

Whey proteins are a by‐product of the cheese industry (Trindade et al., 2019). It could be applied as coating on alginate‐based microcapsule. Whey proteins had different functional properties include gel formation, emulsifier, foam formation, and water bonding (Doherty et al., 2011). Due to biodegradability and widespread use in a variety of foods, whey protein is a good choice for coating (Gbassi et al., 2009).

Xanthan gum is an extracellular polysaccharide and applied as a stabilizer and emulsifier agent in the food and pharmaceutical industries. It could be utilized as a suitable coating on alginate‐based microcapsule surface (Sworn, 2021).

Previous studies have reported that sodium alginate microcapsules coated with whey protein or xanthan gum improve the survival of probiotic bacteria under simulated intestinal gastric conditions (Fareez et al., 2015; Rajam et al., 2012). However, not much is known about the microencapsulation of *L. acidophilus* in sodium alginate and coating with xanthan or/and whey protein as well as inoculation in yogurt. Therefore, the aim of this study was to investigate the effect of coating of *L. acidophilus* microcapsules with whey protein and/or xanthan on the survival of bacteria in yoghurt in a refrigerator for 28 days and under gastrointestinal simulated conditions. In addition, the physicochemical, texture, and sensory properties of yogurt during storage at 4°C for 28 days were investigated.

## 2 MATERIALS AND METHODS

### 2.1 Material

Sodium alginate and xanthan gum were purchased from Sigma‐Aldrich (St‐Louis, USA). MRS agar and broth were bought by Liofilchem (Roseto degli Abruzzi, Italy). Calcium chloride (CaCl_2_), potassium monophosphate, sodium citrate, pepsin, pancreatin, and bile salts were bought from Merck (Darmstadt, Germany). The lyophilized culture of *L. acidophilus* (ATCC 4356) was bought from Persian Type Culture Collection (Tehran, Iran), and low‐fat milk samples (1% fat) were obtained from Damdaran Company (Tehran, Iran), The starter culture containing *Lactobacillus delbrueckii* subsp. *bulgaricus* and *Streptococcus thermophilus* were purchased from Chr. Hansen (Copenhagen, Denmark). Whey protein was obtained from Razan dast Co. (Hamadan, Iran).

### 2.2 ***L*** *actobacillus* ***acidophilus culture activation***

The activation of *L. acidophilus* was performed similar to our previous study (Mousavi et al., 2019). Briefly, the lyophilized culture of *L. acidophilus* was mixed with MRS broth and incubated at 37°C for 24 hr. Then, the tube containing MRS broth and *L. acidophilus* was centrifuged. The supernatant was removed, and normal saline was added to obtain bacteria suspension with opacity equal to the number 2 McFarland standard. The bacterial count in suspension was equal to 6.21 × 10^8^ CFU/mL.

### 2.3 ***Microencapsulation* *and coating of* *L*** *actobacillus* ***acidophilus***

In this study, *L. acidophilus* was microencapsulated in sodium alginate and coated with whey protein and/or xanthan. First, the solution of sodium alginate (1% w/v), whey protein (5.5% w/v), xanthan (0.4% w/v), and calcium chloride (0.1 M) was separately prepared and sterilized at 121°C for 15 min. The microencapsulation of *L. acidophilus* was performed by the extrusion method. One mL of the suspension of *L*. *acidophilus* (containing 6.21 × 10^8^ CFU/mL bacteria) was added into 5 ml of sodium alginate solution and stirred gently for 30 min at 500 RPM. Then, the obtained suspension was added into a calcium chloride solution (0.1 M) by a sterile nozzle syringe to form a bacteria microcapsule. The obtained microcapsules were separated by centrifuging (3,700 g, 5 min) and washing twice with distilled water. Afterward, microcapsules were immersed in 5 ml of coating solution (i.e., whey protein, xanthan, or a mixture both (with ratio of 50:50 v/v) of whey protein and xanthan) and stirred for 30 min at 500 RPM, transferred into a sterile glass container and placed in a freezer (−80°C, 2 hr). In final, it was dried in vacuum‐freeze dryer (operon‐Hwanggeum, Korea) at −60°C for 24h. The coated microcapsule powder was gathered and stored in a refrigerator (4°C).

### 2.4 Calculation of microencapsulation yield

To determine the encapsulation yield, the number of *L. acidophilus* in the coated microcapsule and bacterial suspension was counted. First, 1 g of microencapsulated and/or coated *L. acidophilus* was added into 9 ml of sterile sodium citrate (2% w/v, pH 7) and stirred for 5 min until the capsule was completely dissolved, and the bacteria were released. Second, serial dilutions of bacteria were prepared and cultured on MRS‐agar‐medium‐containing plates by the pour plating method, which were subsequently placed in an incubator at 37°C for 72 hr, and the number of bacteria was counted. On the other hand, the number of bacterial cells in the suspension was measured according to a previous study (Mousavi, Heshmati, Garmakhany, et al., 2019). Then, microencapsulation yield was calculated as follows (Gebara et al., 2013):

Microencapsulation yield (%) = (*N*/*N*_0_) × 100.

Where *N* is the number (log CFU/g) of bacteria released after microencapsulation, and *N*_0_ is the number (log CFU/g) of bacteria in the suspension before microencapsulation and coating.

### 2.5 Determination of capsule properties

The moisture content of the capsules was determined by placing them in an oven at 105 ± 2°C until they attained a constant weight. The surface electrical charge of the capsules (zeta potential) was determined by using a Zeta seizer (Malvern Ltd., Malvern, UK). The volume mean diameter of the microcapsules was measured by using a particle seizer (Malvern Ltd., Malvern, UK).

The water activity of the powders produced by the LabMaster was determined (LabMaster.aw, Novasina, Switzerland).

### 2.6 Preparation of yogurt

In this study, various types of yogurt were produced, including yogurt without a probiotic or control yogurt, (C), yogurt containing free *L. acidophilus* (F), and yogurt containing *L. acidophilus:* microencapsulated in sodium alginate (Alg), microencapsulated in sodium alginate and coated with whey protein (AlgW), microencapsulated in sodium alginate and coated with xanthan (AlgX), and microencapsulated in sodium alginate and coated with whey protein and xanthan (AlgWX). Probiotic bacteria (free, microencapsulated, microencapsulated, and coated) were added along starter culture.

For yogurt production, homogenized and pasteurized milk was heated at 82–86°C for 25 min, followed by cooling to 45°C and pouring into containers, adding the starter culture and probiotic (free, microencapsulated, microencapsulated, and coated), and incubating it at 43–43°C for 3–3.5 hr. Next, samples were transferred into a refrigerator at 4°C for 28 days (Mousavi, Heshmati, Garmakhany, et al., 2019). The viability of *L. acidophilus* as well as the physiochemical, textural, and sensory properties of the product were investigated after 14 and 28 days of storage in a refrigerator (4°C).

### 2.7 Preparation of simulated gastric and intestinal juice

Simulated gastric juice (SGI) was prepared by the addition of 0.3 g of pepsin into 100 ml of a sodium chloride solution (0.2% w/v) and adjustment of the pH to 2.0 using 0.1 N HCl (Chaikham, 2015). Simulated intestinal juice (SIJ) was prepared by the suspension of pancreatic (0.1% w/v) and bile salts (0.45% w/v) into sodium citrate (2%) and adjustment of the pH to 7.4 using 0.1 N NaOH (Fazilah et al., 2019). Both SGJ and SIJ were filtered through a membrane (0.45 µm, Millipore, Spain) for sterilization.

### 2.8 Viability of Lactobacillus acidophilus in yogurt after exposure to simulated gastrointestinal conditions

First, 1 g of yogurt samples that were stored in the refrigerator for 1, 14, and 28 days was placed in tubes containing 9 ml of SGJ and incubated at 37°C for 120 min. Second, the pH of the samples was adjusted to 7, and 10 ml of SIJ was added, followed by incubation at 37°C for 300 min. Finally, the number of *L. acidophilus* was counted according to the aforementioned method.

### 2.9 pH and acidity of yogurt

The pH of the yogurt samples was measured using a pH meter (Denver Instruments, TX, USA).

The acidity of yogurt samples was measured by the titration method and reported according to the percentage of lactic acid (Hasani et al., 2017).

### 2.10 Syneresis measurement of yogurt

First, 5 g of yogurt samples was poured on a filter paper placed on a funnel in a glass container in a refrigerator at 5°C for 2 hr. Second, the amount of liquid collected at the bottom of the container was weighed. Syneresis was calculated by the following equation:

Syneresis = (weight of the collected liquid/the initial weight of yogurt) × 100.

### 2.11 Texture analysis

Textural properties such as firmness, cohesiveness, adhesiveness, and gumminess, as well viscosity, were determined using a Zwick Texture Analyzer (Roller Company, Ulm, Germany). Extrusion test and a cylindrical probe (diameter of 40 mm) were applied for texture assessment. A cylindrical‐type probe with a diameter of 40 mm was used. The penetration speed and depth of the probe into the yogurt were 10 mm/s and 25 mm, respectively.

### 2.12 Sensory evaluation

Fifty trained panelists examined the sensory properties of samples, including taste, mouthfeel, appearance, and overall acceptability, by a 5‐point hedonic scale. The highest (5) and lowest (1) scores revealed that the sample is very good and very bad, respectively (Mousaviet al., 2019). The samples were presented to panelists at a temperature of 4°C.

### 2.13 Statistical analysis

All experiments were performed in triplicate. The mean ±standard deviation of data was presented. One‐way ANOVA and Duncan's multiple range tests were employed for data analysis by SPSS version 22.0 Advanced Statistics (IBM, Armonk, NY, USA). The significant level was considered to be *p* < .05.

## 3 RESULTS AND DISCUSSION

### 3.1 Microencapsulation yield and physico‐chemical properties of coated *Lactobacillus acidophilus* microcapsule

The results for the microencapsulation yields of *L*. *acidophilus* microcapsules uncoated/coated with whey protein and/or xanthan are shown in Table 1. The microencapsulation yield is an important factor for assessing the effective encapsulation method and indicating the efficiency of the chosen encapsulating agents (Bora et al., 2019). The results revealed that coating increased microencapsulation yields. The highest microencapsulation yield (99.81 ± 1.03%) was found in the microcapsules coated with whey protein and xanthan. Some factors such as the hydrogen bonding between carboxylate groups of sodium alginate and xanthan and good interaction between sodium alginate (negative charge) and whey protein (positive charge) and freeze‐drying method could increase microencapsulation yield (Bekhit et al., 2016; Gbassi & Vandamme, 2012). The previous study indicated the freeze‐drying method had a higher yield than the spray‐drying method (Rajam et al., 2012).

**TABLE 1:** Encapsulation efficiency and physico‐chemical properties of encapsulation and coated *L. acidophilus*

Treatment	Microencapsulation efficiency (%)	Diameter (µm)	Zeta potential (mV)	Moisture (%)	aw
Alg	77. 9 ± 1.25^d^	37.39 ± 1.23^d^	−15.8±0.87^b^	14.08 ± 1.21^a^	0.24 ± 0.02^a^
AlgW	91.99 ± 2.21^c^	41.88 ± 1.41^c^	−10.8±0.65^a^	11.81 ± 1.24^b^	0.19 ± 0.01^b^
AlgX	95.94 ± 2.85^b^	48.35 ± 0.89^b^	−16.3 ± 1.05^b^	9.09 ± 0.21^c^	0.16 ± 0.03^c^
AlgWX	99.81 ± 1.03^a^	52.01 ± 1.25^a^	−9.21 ± 0.65^a^	7.74 ± 0.98^d^	0.12 ± 0.02^d^

Note

Values are expressed as the mean ± *SD* of three determinations. Means followed by different lowercase letters differ statistically within each column (*p* < .05).

F: yogurt containing free (without microencapsulated) *L. acidophilus*, Alg: yogurt containing *L. acidophilus* microencapsulated with sodium alginate; AlgW: yogurt containing *L. acidophilus* microencapsulated with sodium alginate and coated with whey protein; AlgX: yogurt containing *L. acidophilus* microencapsulated with sodium alginate and coated with xanthan; AlgWX: yogurt containing *L. acidophilus* microencapsulated with sodium alginate and coated with whey protein and xanthan.

The coating of microcapsules with whey protein and/or xanthan increased the diameter of the *L. acidophilus* microencapsulated in sodium alginate (ranging from 37.39 ± 1.23 µm to 52.01 ± 1.25 µm). The microcapsule requires a diameter of 40–100 µm to obtain suitable survival of the microencapsulated probiotic without affecting the foods’ sensory properties (Xia et al., 2020). The diameter depends mainly on the size of the syringe, the microencapsulation method, the viscosity of the solution, and the distance between the syringe and the calcium chloride solution (Nualkaekul et al., 2012).

The range of zeta potential values of the microcapsules prepared in this study was from −9.21 ± 0.65 to −16.3 ± 1.05 mV (Table 1). The high value of the colloidal particles’ zeta potential increases the electrostatic repulsion force and the physical stability of the system. Various factors such as pH, ionic strength, type and concentration of polysaccharides, and used protein macromolecules (and the ratio between these) all affect the level of surface charge and zeta potential (Ding et al., 2019).

The microcapsules’ aw and moisture levels ranged from 0.12–0.24 and 7.74%–14.08%, respectively. The moisture content of the probiotic microcapsules is an important factor in protecting bacteria during storage. Microorganisms have better survival rates in low‐moisture environments due to reduced biochemical reactions (Peredo et al., 2016). In the present study, the coating decreased aw and moisture levels of the probiotic microcapsules. The microcapsules’ aw was lower than the limit (<0.6) recommended for microbiological stability (Ashwar et al., 2018); it was also similar to a study by Peredo et al., (2016). Here, the authors reported aw ranging from 0.18 to 0.23 for *Lactobacillus casei* encapsulated with potato starch–alginate, psyllium–alginate, or inulin–alginate.

### 3.2 ***L*** *actobacillus* ***acidophilus*** viability

**FIGURE 1:**
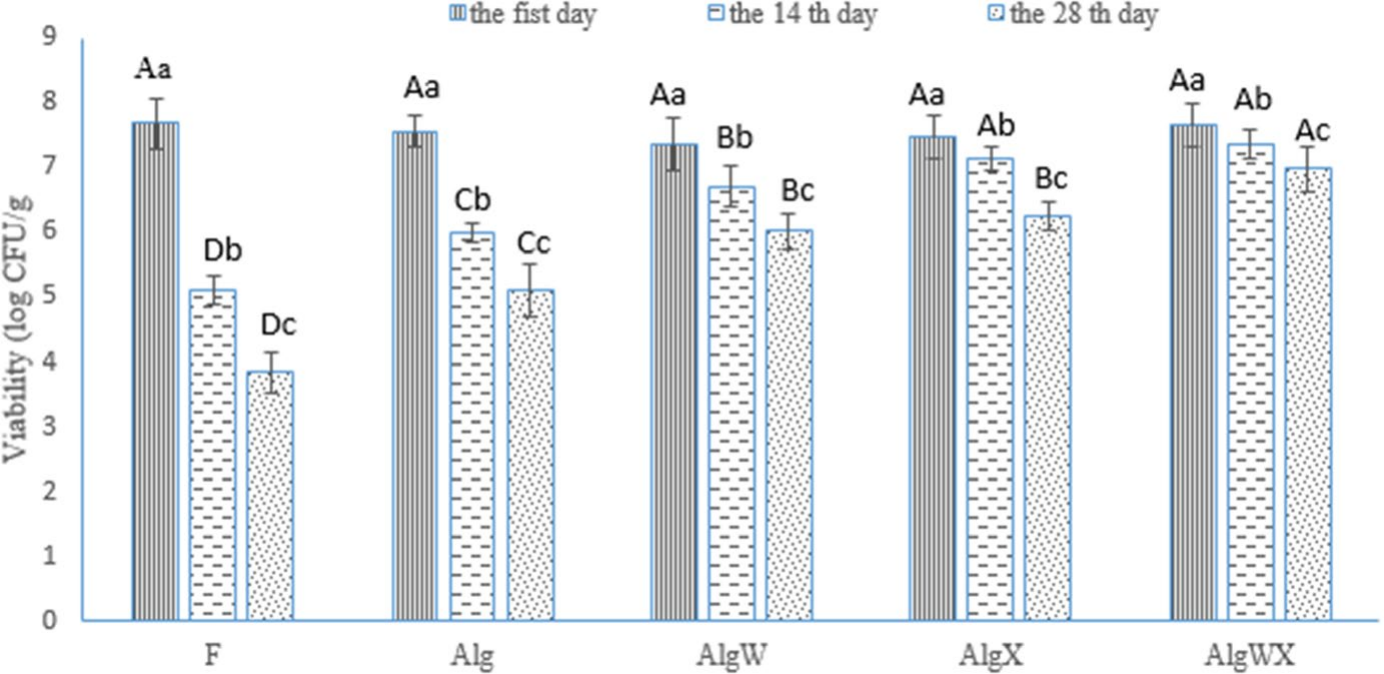
The number of viable cells of *L. acidophilus* (log CFU/g) in probiotic yoghurt after 1, 14 and 28 days of storage in refrigerator (*n* = 3). F: yogurt containing free (without microencapsulated) *L. acidophilus*, Alg: yogurt containing *L. acidophilus* microencapsulated with sodium alginate; AlgW: yogurt containing *L. acidophilus* microencapsulated with sodium alginate and coated with whey protein; AlgX: yogurt containing *L. acidophilus* microencapsulated with sodium alginate and coated with xanthan; AlgWX: yogurt containing *L. acidophilus* microencapsulated with sodium alginate and coated with whey protein and xanthan. Uppercase letters above columns indicate significant difference (*p* <.05) among different treatments for the same storage period. Lowercase letters above columns indicate significant difference (*p* <.05) among different storage period for each treatment

**FIGURE 2:**
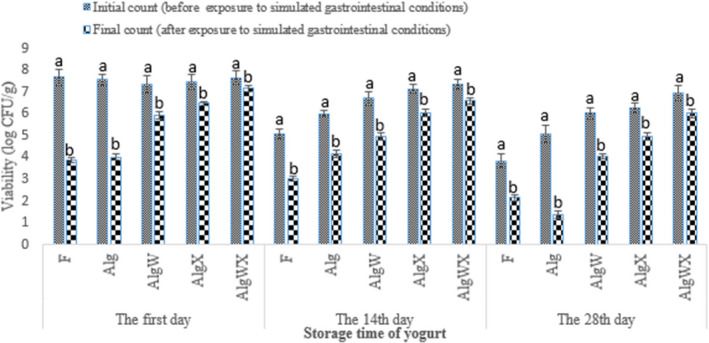
Change in the number of viable cells of free, microencapsulated, and or coated *L. acidophilus* (log CFU/g) in yogurt after exposure to simulated gastrointestinal conditions (*n* = 3). F: yogurt containing free (without microencapsulated) *L. acidophilus*, Alg: yogurt containing *L. acidophilus* microencapsulated with sodium alginate; AlgW: yogurt containing *L. acidophilus* microencapsulated with sodium alginate and coated with whey protein; AlgX: yogurt containing *L. acidophilus* microencapsulated with sodium alginate and coated with xanthan; AlgWX: yogurt containing *L. acidophilus* microencapsulated with sodium alginate and coated with whey protein and xanthan. Lowercase letters above columns indicate significant difference (*p* <.05) between initial and final count of *L. acidophilus* of each treatment

**FIGURE 3:**
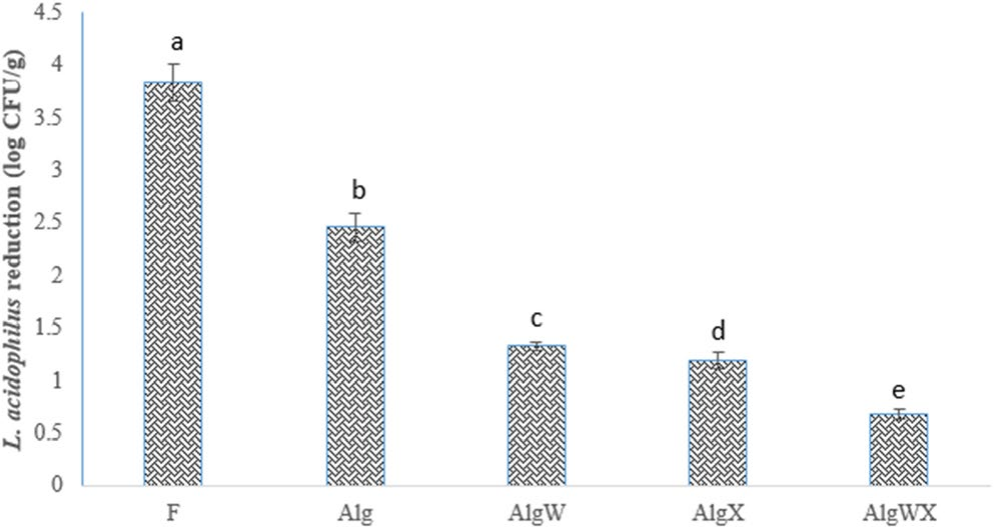
The reduction value of viable cells of *L. acidophilus* (log CFU/g) in probiotic yoghurt after 28 days of storage in refrigerator (*n* = 3). F: yogurt containing free (without microencapsulated) *L. acidophilus*, Alg: yogurt containing *L. acidophilus* microencapsulated with sodium alginate; AlgW: yogurt containing *L. acidophilus* microencapsulated with sodium alginate and coated with whey protein; AlgX: yogurt containing *L. acidophilus* microencapsulated with sodium alginate and coated with xanthan; AlgWX: yogurt containing *L. acidophilus* microencapsulated with sodium alginate and coated with whey protein and xanthan. The various letters above columns indicate significant difference (*p* <.05) among different treatments

**FIGURE 4:**
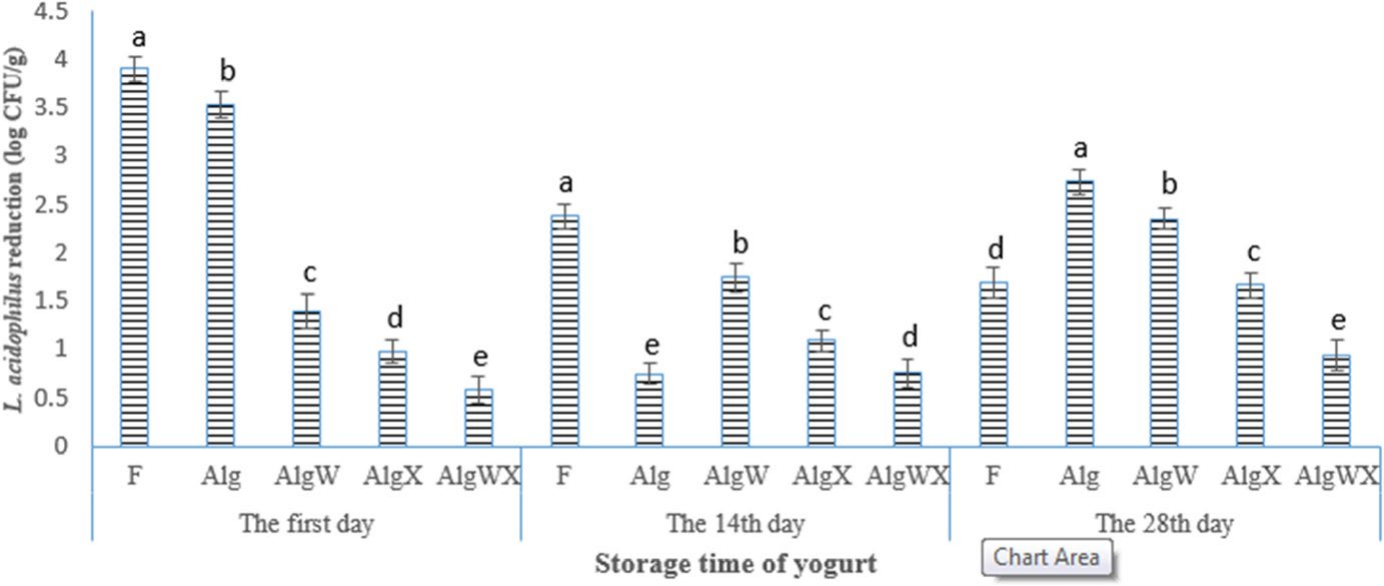
The reduction value of viable cells of *L. acidophilus* (log CFU/g) in probiotic yoghurt after 1, 14 and 28 days of storage in refrigerator and exposure to simulated gastrointestinal conditions (*n* = 3). The various letters above columns indicate significant difference (*p* <.05) among different treatments for a particular day of the storage period

In comparison with free bacteria, microencapsulation and coating could significantly increase the viability of *L. acidophilus* in yogurt during storage and after exposure to simulated gastrointestinal conditions (Figures 1, 2, 3, 4). The reduction in *L. acidophilus* as free, microencapsulated with Alg, microencapsulated and coated with AlgW, AlgX, and AlgWX after 28 days of yogurt storage was 3.84, 2.46, 1.33, 1.2, and 0.68 log CFU/g, respectively (Figure 3). Figure 4 illustrates the reduction in *L. acidophilus* in yogurt after various days of storage and exposure to simulated gastrointestinal conditions. After 28 days of storage of yogurt in a refrigerator and exposure to simulated gastrointestinal conditions, the reduction value of *L. acidophilus* as free; microencapsulated with Alg; microencapsulated and coated with AlgW, AlgX, and AlgWX was 5.63, 6.2, 3.29, 2.47, and 1.63 log CFU/g, respectively (Figure 3). Thus, the coating with whey protein and/or xanthan intensified the viability and reduced the death rate of the probiotic. The coating caused pore size of sodium alginate microcapsule was decreased. Yao et al., (2020) declared the pore size of alginate‐based microcapsule affects the survival of probiotics under adverse conditions. For the survival of bacteria in the gastrointestinal tract, the pore size of microcapsule should be smaller than the size of hydrogen ions (<1 nm) and enzymes (<5 nm) (Yao et al., 2020). At the end of the expiration date (day 28), all the yogurt samples containing coated *L. acidophilus* had viable probiotic counts higher than the recommend (6 log CFU/g) level (Nyanzi et al., 2021). The highest probiotic count (6.98 log CFU/g) after 28 days was found in the sample containing *L. acidophilus* microencapsulated with sodium alginate and coated with whey protein and xanthan.

The reduction level of the probiotic coated with whey protein and/or xanthan in the current study was similar to study done by Chen et al., (2017). The authors found *Bifidobacterium* reduction value (after 21 day) in the sample of yogurt containing free bacteria and bacteria encapsulated with xanthan–chitosan and xanthan–chitosan–xanthan were 0.96 and 1.23 log CFU/mL, respectively. In another study, the reduction in *L. acidophilus* coated with alginate, chitosan, and galacto‐oligosaccharide after 28 days of storage at 4°C was 1.8 log CFU/mL (Krasaekoopt & Watcharapoka, 2014). The reduction in *L. acidophilus* coated with pectin‐whey protein and inoculated into yogurt (after 35 days of storage) was 0.2 log CFU/g (Ribeiro et al., 2014). The count of *L. plantarum* coated with alginate and chitosan in yogurt after 35 days of storage decreased to 0.55 log CFU/mL (Brinques & Ayub, 2011).

### 3.3 Physiochemical properties of yogurt

In the present study, a significant difference between pH of yogurt containing coated and microencapsulated *L. acidophilus* and contro yogurt was not observed. However, yogurt containing free *L. acidophilus* had lower pH and showed a significant difference (*p* < .05) compared with control yogurt and yogurt containing microencapsulated and/or coated *L. acidophilus*. Similar to our finding, Prasanna and Charalampopoulos (2019) found no difference between the pH of control yogurt and yogurt containing *Bifidobacterium* coated in an alginate–goat milk–inulin matrix. In all the treatments, the pH increased during the storage period due to lactic acid production by starter culture. The yogurt samples containing free *L. acidophilus* stored for 28 days in a refrigerator had the lowest pH (2.49 ± 0.28).

In the present study, the acidity of the yogurt samples significantly increased during storage (Table 2). The inoculation of *L. acidophilus* as microencapsulated and/or coated could not have caused considerable changes in the acidity compared with control sample. However, these samples had lower acidity than yogurt containing free *L. acidophilus*. Similar to our finding, Chen et al., 2017 indicated that the acidity of yogurt containing *Bifidobacterium* encapsulated with xanthan–chitosan and xanthan–chitosan–xanthan was lower than the acidity of the samples containing free probiotics. The microencapsulation and coating process decreased the activity of probiotic and postacidification in yogurt. Therefore, pH and acidity of samples containing *L. acidophilus* as microencapsulated and/or coated had not significant difference with the control sample, while free *L. acidophilus* had higher activity and caused lower pH and greater acidity (Ribeiro et al., 2014).

**TABLE 2:** Chemical properties of probiotic yogurt containing free and microencapsulated and coated *L. acidophilus* during yogurt storage at 4°C

Parameter	Storage time (day)	Treatment					
		C	F	Alg	AlgW	AlgX	AlgWX
pH	1	4.32 ± 0.39^Aa^	4.29 ± 0.39^Aa^	4.35 ± 0.39^Aa^	4.28 ± 0.39^Aa^	4.34 ± 0.25^Aa^	4.23 ± 0.16^Ab^
	14	4.25 ± 0.37^Aa^	3.54 ± 0.35^Bab^	4.11 ± 0.37^Aa^	3.97 ± 0.37^Aab^	4.20 ± 0.24^Aab^	4.08 ± 0.15^Aab^
	28	3.81 ± 0.3^Ab^	2.49 ± 0.28^Bc^	3.33 ± 0.31^Ab^	3.55 ± 0.33^Ab^	3.57 ± 0.21^Ab^	3.66 ± 0.14^Ab^
Acidity	1	1.11 ± 0.1^Ab^	1.14 ± 0.1^Ac^	1.08 ± 0.1^Ac^	1.13 ± 0.1^Ab^	1.10 ± 0.06^Aa^	1.08 ± 0.04^Ab^
	14	1.13 ± 0.11^Aab^	1.31 ± 0.12^Aab^	1.24 ± 0.11^Aab^	1.29 ± 0.11^Aab^	1.20 ± 0.07^Aab^	1.24 ± 0.05^Aa^
	28	1.23 ± 0.12^Ba^	1.57 ± 0.13^Aa^	1.39 ± 0.12^Ba^	1.33 ± 0.12^Ba^	1.27 ± 0.07^Ba^	1.25 ± 0.05^Ba^
Syneresis	1	15.98 ± 0.97^Ab^	18.25 ± 1.16^Ab^	14.25 ± 0.84^Aa^	8.96 ± 0.33^Ba^ 0	039 ± 0.31^Ca^	0.38 ± 0.19^Ca^
	14	22.11 ± 1.33^Aa^	25.24 ± 0.96^Aa^	0	0.58 ± 0.02^Bb^	0	0
	28	26.18 ± 1.59^Aa^	30.45 ± 1.14^Aa^	0	0	0	0

Note

Values are expressed as the mean ± *SD* of three determinations. Means followed by different uppercase letters differ statistically within each row (*p* < .05). Means followed by different lowercase letters differ statistically within each column (*p* < .05).

C: control yogurt (without probiotic), F: yogurt containing free (without microencapsulated) *L. acidophilus*, Alg: yogurt containing *L. acidophilus* microencapsulated with sodium alginate; AlgW: yogurt containing *L. acidophilus* microencapsulated with sodium alginate and coated with whey protein; AlgX: yogurt containing *L. acidophilus* microencapsulated with sodium alginate and coated with xanthan; AlgWX: yogurt containing *L. acidophilus* microencapsulated with sodium alginate and coated with whey protein and xanthan.

### 3.4 Syneresis

The highest syneresis value was related to yogurt containing free *L. acidophilus*. When *L. acidophilus* was microencapsulated in alginate and inoculated into the yogurt, this resulted in syneresis reduction (Table 2). The coating of whey protein and xanthan on *L. acidophilus* microcapsule could significantly decrease syneresis of yogurt, so that the lowest syneresis value was observed in yogurt containing *L. acidophilus* microcapsule coated with whey protein and xanthan (Table 2). The impact of xanthan on syneresis reduction was higher than whey protein. The components applied for probiotic microencapsulation and coating including sodium alginate, whey protein, and xanthan absorbed water and could decrease syneresis. The role of protein‐based compounds and fibers on syneresis reduction was previously documented (Mousavi, Heshmati, Daraei Garmakhany, et al., 2019). In control yogurt and yogurt containing free *L. acidophilus,* syneresis was increased during storage. However, no syneresis was observed in samples containing *L*. *acidophilus* microcapsule coated with whey protein and/or xanthan.

### 3.5 The viscosity of yogurt

The viscosity of yogurt samples depended on storage time and coating type of inoculated *L. acidophilus* (Table 3). In all samples during the storage period, viscosity was increased. There was no significant difference between the viscosity of control yogurt and yogurt containing free *L. acidophilus,* although coating and microencapsulation of *L. acidophilus* caused yogurt viscosity to increase. The highest viscosity (43,053.34 ± 5,321.87 centipoise) was related to yogurt containing *L. acidophilus* microcapsule coated with whey protein and xanthan and stored for 28 days in a refrigerator. Xanthan and sodium alginate are an anionic hydrocolloid and can interact with positive charge particles within milk including proteins or calcium ions. These interactions strengthen the protein network and result in an increase in viscosity (Macit et al., 2019). In addition, the increase in whey protein concentration in yogurt enhanced the interactions of inter‐particles, such as protein and fat globules, and improved viscosity (Krzeminski et al., 2011). In a previous study performed by Afzaal et al., (2019), results indicated that the coating of *L. acidophilus* with carrageenan enhanced yogurt viscosity. These findings were similar to our results.

**TABLE 3:** Viscosity and texture properties of probiotic yogurt containing free, microencapsulated and coated *L. acidophilus* during yogurt storage at 4°C

Parameter	Storage time (day)	Treatment					
		C	F	Alg	AlgW	AlgX	AlgWX
Viscosity (centipoise)	1	8,894.37 ± 1937.4^Eb^	8,959.26 ± 164.63^Eb^	10,524.15 ± 152.42^dc^	12,916.02 ± 128.75^Cc^	16,742.97 ± 220.5^Bc^	19,374.23 ± 253.2^Ac^
	14	9,068.67 ± 226.3^Eb^	9,068.67 ± 251.24^Eb^	11,241.71 ± 112.17^Db^	17,460.52 ± 293.02^Cb^	23,679.34 ± 293.02^Bb^	21,287.48 ± 300.77^Ab^
	28	12,742.97 ± 265.96^Ea^	12,613.19 ± 411.99^Ea^	19,091.56 ± 200.16 Da	20,330.74 ± 386.84^Ca^	36,595.34 ± 466.11^Ba^	43,053.34 ± 321.87^Aa^
Firmness (*N*)	1	0.44 ± 0.04^Cb^	0.50 ± 0.09^Cb^	0.58 ± 0.12^Cc^	0.54 ± 0.09^Cc^	0.75 ± 0.01^Bc^	0.87 ± 0.08^Ac^
	14	0.47 ± 0.05^Cb^	0.64 ± 0.11^Cab^	0.95 ± 0.13^Bb^	0.94 ± 0.13^Bb^	1.12 ± 0.14^Bb^	1.25 ± 0.01^Ab^
	28	0.85 ± 0.09 Da	0.82 ± 0.18 Da	1.06 ± 0.24^Ca^	1.10 ± 0.17^Ca^	1.64 ± 0.21^Ba^	1.95 ± 0.18^Aa^
Adhesiveness (*N*)	1	0.25 ± 0.36^Ca^	0.22 ± 0.01^Ca^	0.23 ± 0.02^Ca^	0.27 ± 0.21^Ba^	0.29 ± 0.15^Ba^	0.35 ± 0.31^Aa^
	14	0.21 ± 0.02^Bb^	0.21 ± 0.02^Ba^	0.20 ± 0.03^Bb^	0.26 ± 0.02^Aa^	0.27 ± 0.02^Ab^	0.28 ± 0.02^Ab^
	28	0.12 ± 0.02^Cc^	0.14 ± 0.01^Ca^	0.15 ± 0.01^Cb^	0.17 ± 0.03^Bb^	0.21 ± 0.02^Ab^	0.23 ± 0.01^Ab^
Cohesiveness (*N*)	1	0.53 ± 0.05^Ac^	0.52 ± 0.07^Ab^	0.55 ± 0.08^Ab^	0.41 ± 0.07^Bb^	0.44 ± 0.04^Ba^	0.32 ± 0.04^Bb^
	14	0.62 ± 0.06^Ab^	0.58 ± 0.07^Aa^	0.61 ± 0.08^Aa^	0.40 ± 0.07^Bb^	0.46 ± 0.07^Ba^	0.44 ± 0.07^Ba^
	28	0.67 ± 0.06^Aa^	0.59 ± 0.08^Ba^	0.62 ± 0.09^Ba^	0.46 ± 0.08^Ca^	0.47 ± 0.07^Ca^	0.45 ± 0.07^Ca^
Gumminess (*N*)	1	0.38 ± 0.06^Ab^	0.4 ± 0.06^Ab^	0.35 ± 0.05^Ab^	0.37 ± 0.06^Aa^	0.37 ± 0.05^Ab^	0.27 ± 0.03^Aa^
	14	0.57 ± 0.09^Aab^	0.51 ± 0.07^Aab^	0.44 ± 0.07^Ab^	0.49 ± 0.07^Aa^	0.47 ± 0.06^Aab^	0.30 ± 0.03^Aa^
	28	0.68 ± 0.1^Aa^	0.69 ± 0.11^Aa^	0.81 ± 0.11^Aa^	0.53 ± 0.09^Aa^	0.73 ± 0.1^Aa^	0.57 ± 0.06^Aa^

Note

Values are expressed as the mean ± *SD* of three determinations. Means followed by different uppercase letters differ statistically within each row (*p* < .05). Means followed by different lowercase letters differ statistically within each column (*p* < .05).

C: control yogurt (without probiotic), F: yogurt containing free (without microencapsulated) *L. acidophilus*, Alg: yogurt containing *L. acidophilus* microencapsulated with sodium alginate; AlgW: yogurt containing *L. acidophilus* microencapsulated with sodium alginate and coated with whey protein; AlgX: yogurt containing *L. acidophilus* microencapsulated with sodium alginate and coated with xanthan; AlgWX: yogurt containing *L. acidophilus* microencapsulated with sodium alginate and coated with whey protein and xanthan.

### 3.6 Texture properties of yogurt

Firmness is the most important parameter for evaluating yogurt texture and indicating hardness. It is considered as the force required to achieve a certain deformation (Mudgil et al., 2017). There was no significant difference between the firmness of control yogurt and yogurt containing free *L. acidophilus*. Thus, this showed that the addition of a probiotic did not impact firmness (Table 3). However, samples containing microencapsulated and coated *L. acidophilus* had higher firmness. The highest firmness value (0.1985 ± 0.18 N) was observed in the yogurt sample containing *L*. *acidophilus* microcapsule coated with whey protein and xanthan. In the present study, the firmness value of all samples was increased during the storage period. In a previous study performed by Mousa et al., (2014), the firmness of yogurt containing *Bifidobacterium bifidum* microencapsulated with whey protein and sodium alginate was raised gradually and then reduced after the 14th day. The authors concluded that firmness reduction was related to postacidification and enzymatic activity of lactic acid bacteria.

The coating of *L. acidophilus* microcapsule with whey protein and xanthan resulted in adhesiveness reduction and cohesiveness increase in yogurt. Adhesiveness is the force value one needs to separate the material that sticks to the mouth during eating, while cohesiveness demonstrates the power of internal bonds constituting the body and structure of food. It is the force value for creating substance deformation without breaks (Kose et al., 2018). High adhesiveness could result in a higher degree of yogurt stickiness in the mouth, which is a negative attribute. Lower values of adhesiveness are more acceptable for consumers (Li et al., 2017). There is an inverse relationship between adhesiveness and firmness (Mani‐López et al., 2014). In all samples during storage, adhesiveness was decreased, while cohesiveness increased. These findings were similar to previous studies (Akalın et al., 2012; Mousavi, Heshmati, Garmakhany, et al., 2019). It seems that whey protein and xanthan applied as a coating for *L. acidophilus* absorbed water, thus causing an increase in adhesiveness and a decline in cohesiveness of yogurt.

Gumminess is the force needed to break down a semi‐solid material, so it can be ready to swallow. Gumminess depended on firmness and cohesiveness attributes. There was no significant difference between the gumminess of various samples. However, the gumminess value of each sample was increased during storage, which is a finding similar to a previous study (Pinto et al., 2017).

### 3.7 Sensory properties of yogurt

No significant difference in the taste, mouthfeel, appearance, and overall acceptability for yogurt samples containing free and microencapsulated and/or coated *L. acidophilus* was observed (Table 4). One of the criteria's for probiotic selection is the strains selected inoculated into yogurt should not have a negative impact on sensory quality (Nyanzi et al., 2021), and our finding showed that used probiotic had this property.

**TABLE 4:** Sensory properties of probiotic yogurt containing free and microencapsulated and coated *Lactobacillus acidophilus* during yogurt storage at 4°C

Treatment	Taste	Mouthfeel	Appearance	Overall acceptability
C	4.75 ± 0.55	4.82 ± 0.57	5.00 ± 0.60	5.00 ± 0.58
F	4.72 ± 0.47	4.52 ± 0.54	4.80 ± 0.58^Ab^	4.14 ± 0.49
Alg	4.8 ± 0.58	4.60 ± 0.55	4.80 ± 0.58	4.90 ± 0.59
AlgW	4.75 ± 0.57	4.50 ± 0.56	4.75 ± 0.57	4.81 ± 0.58
AlgX	4.70 ± 0.56	4.70 ± 0.56	4.8 ± 0.57	4.92 ± 0.58
AlgWX	4.40 ± 0.44	4.60 ± 0.46	4.50 ± 0.45	4.63 ± 0.46

Note

Values are expressed as the mean±*SD* of three determinations. There are no significant difference in the taste, mouthfeel, appearance, and overall acceptability for various yogurt samples

C: control yogurt (without probiotic), F: yogurt containing free (without microencapsulated) *L. acidophilus*, Alg: yogurt containing *L. acidophilus* microencapsulated with sodium alginate; AlgW: yogurt containing *L. acidophilus* microencapsulated with sodium alginate and coated with whey protein; AlgX: yogurt containing *L. acidophilus* microencapsulated with sodium alginate and coated with xanthan; AlgWX: yogurt containing *L. acidophilus* microencapsulated with sodium alginate and coated with whey protein and xanthan.

Our results were similar to those reported by Ribeiro et al., (2014). These authors evaluated sensory properties of yogurt samples containing *L. acidophilus* as free or microencapsulated with pectin and whey protein after storage for 1 and 35 days and reported no difference between the taste, aroma, appearance, and overall acceptability of these samples compared with the control specimen (Ribeiro et al., 2014). However, Afzaal et al., (2019) have reported a significant difference between the sensory attributes of control samples and yogurt containing *L. acidophilus* encapsulated in sodium alginate and carrageenan.

## 4 CONCLUSION

In this study, the use of whey protein and xanthan as a coating on *L*. *acidophilus* microcapsule could lead to the considerable increase in the viability of this probiotic in yogurt during storage and in simulated gastrointestinal conditions. In addition, the applied coating did not cause any significant change in the pH and acidity, sensory attributes, and gumminess of yogurt, but it simultaneously increased the firmness, adhesiveness, and viscosity of this product and caused a significant decrease in syneresis and cohesiveness. Therefore, the application of coating of xanthan and/or whey protein on *L*. *acidophilus* microcapsule surface is a suitable method for increasing the viability of this bacteria in yogurt while simultaneously improving the physico‐chemical and textural properties of yogurt; in addition, adverse effects on the sensory attributes of the product were not observed.

## 5 STUDIES INVOLVING HUMAN SUBJECTS

Human subjects for this study were approved by the Research Ethics Committee of Hamadan University of Medical Sciences, Hamadan, Iran for the Protection of Human Subjects in Research (IRB) protocol IR.UMSHA.REC.1398.270.

## 6 STUDIES INVOLVING ANIMAL OR HUMAN SUBJECTS

Protocols and procedures utilized in this study for sensory evaluation of samples by human panelists were ethically approved by Research Ethics Committee of Hamadan University of Medical Sciences, Hamadan (IR.UMSHA.REC.1398.270).

## CONFLICT OF INTEREST

The authors declare that they do not have any conflict of interest.

## AUTHORS CONTRIBUTION

**Mina Khorshidi:** Data curation (equal); Methodology (equal); Writing‐original draft (equal). **Ali Heshmati:** Formal analysis (equal); Project administration (lead); Supervision (equal); Validation (equal); Writing‐original draft (equal); Writing‐review & editing (equal). **Mehdi Taheri:** Data curation (equal); Project administration (supporting); Supervision (equal). **Mostafa Karami:** Data curation (equal); Writing‐original draft (equal). **Reza Mahjub:** Data curation (equal); Methodology (equal); Supervision (equal).
